# Forest Soil pH and Dissolved Organic Matter Aromaticity Are Distinct Drivers for Soil Microbial Community and Carbon Metabolism Potential

**DOI:** 10.1007/s00248-025-02493-5

**Published:** 2025-01-27

**Authors:** Zongxiao Zhang, Qiang Zhang, Xue Guo, Zhenzhong Zeng, Yinghui Wang, Peng Zhang, Dengzhou Gao, Guisen Deng, Guodong Sun, Yuanxi Yang, Junjian Wang

**Affiliations:** 1 State Environmental Protection Key Laboratory of Integrated Surface Water-Groundwater Pollution Control, School of Environmental Science and Engineering, Southern University of Science and Technology, Shenzhen, 518055 Guangdong China; 2 Guangdong Provincial Key Laboratory of Soil and Groundwater Pollution Control, Southern University of Science and Technology, Shenzhen, 518055 Guangdong China; 3 School of Environment, Harbin Institute of Technology, Harbin, 150090 China; 4 State Key Joint Laboratory of Environment Simulation and Pollution Control, School of Environment, Tsinghua University, Beijing, 100089 China; 5 Key Laboratory of Humid Subtropical Eco-Geographical Process of Ministry of Education, College of Geographical Sciences, Fujian Normal University, Fuzhou, 350000 China

**Keywords:** Chinese forest soil, Microbial co-occurrence, Ecological niche, Dissolved organic matter, Carbon metabolic function

## Abstract

The ecological niche separation of microbial interactions in forest ecosystems is critical to maintaining ecological balance and biodiversity and has yet to be comprehensively explored in microbial ecology. This study investigated the impacts of soil properties on microbial interactions and carbon metabolism potential in forest soils across 67 sites in China. Using redundancy analysis and random forest models, we identified soil pH and dissolved organic matter (DOM) aromaticity as the primary drivers of microbial interactions, representing abiotic conditions and resource niches, respectively. Our network comparison results highlighted significant differences in microbial interactions between acidic and non-acidic soils, suggesting the critical influences of abiotic conditions on microbial interactions. Conversely, abiotic resource niches played a more pivotal role in shaping the carbon metabolism of soil microbes, supporting the concept that resource niche-based processes drive microbial carbon cycling. Additionally, we demonstrated that microbial interactions contributed significantly to ecosystem function stability and served as potential ecological indicators of microbial functional resilience under environmental stress. These insights emphasize the critical need to preserve microbial interactions for effective forest ecosystem management and projection of ecological outcomes in response to future environmental changes.

**Supplementary Information:**

The online version contains supplementary material available at 10.1007/s00248-025-02493-5.

## Introduction

Microbial interactions are crucial for the soil biogeochemical functioning in forest ecosystems [1]. Molecular ecological network (MEN) analysis is a powerful tool based on microbial DNA sequencing results. By constructing interaction networks among microorganisms, it reveals the interactions and coexistence patterns within microbial communities, providing new insights into the understanding of microbial ecology [2–4]. Within the MEN, a network node represents specific taxonomic groups, and a network edge in the network symbolizes observed abundance correlations between these taxa, thereby enabling inference regarding the characteristics of biotic relationships [5]. MEN properties can reflect the modularity, sparsity, and robustness of the network and have been effectively utilized for predicting the complexity and stability of microbial relationships [4, 6, 7], which is crucial for maintaining functional stability within the microbial community [8]. Properties of MENs provide insights into the centrality and functional importance of microbial species in regulating community structure and stability. For example, node degree represents the number of direct connections a microorganism has within the network [2]. Additionally, modularity refers to the degree to which the network is divided into distinct modules; nodes within each module are strongly interconnected, while nodes between different modules exhibit limited connectivity [9]. Generally, higher complexity and robust ecological networks indicate that microbial relationships are more stable, enhancing resistance to environmental stressors or changes [10].

Ecological niche theory is the fundamental framework for understanding or forecasting the distribution and diversity of microbial communities in natural environments [11, 12]. Ecological niche types serve as crucial environmental filters or stressors, shaping the composition of microbial communities and ascertaining whether a congregation of microbial taxa or the operational taxonomic units (OTU) is well-suited to flourish at a specific locale, in the present, the past, or the future [13]. Thus, ecological niche theory could address the fundamental ecological issue of species-environment relationships, for example, how environmental filtering and resource competition shape the composition and function of microbial communities. Delineating the environmental niches of the microbe species can be employed to investigate the primary environmental filtering factors influencing the spatial distribution characteristics of microbe [14]. Soil variations that drive the environmental filtering process in microbial communities can be divided into abiotic resources and abiotic conditions. The abiotic resources are consumed by microbial communities, and microbial species may compete for their limitation, while abiotic conditions are tolerated but not competed for [12]. Resource competition is crucial for the coexistence of microbial species, as it determines their ability to acquire and utilize limited resources. If the competition for resources is crucial for the coexistence of microbial species, the community relationships of microbial species can exhibit evident dynamics along the resource niche dimensions. Conversely, if the environmental conditions (such as soil pH and temperature) significantly impact the composition or distribution of microbial communities, the co-existing species change significantly along the condition niche dimensions [15]. Thus, the distinction between the niche dimensions associated with resources and those reflecting conditions is crucial for understanding and predicting the impact of environmental filtering on microorganisms [16, 17].

Although a wealth of forest soil environmental data is available, our current understanding of how soil niche type separation influences microbial coexistence remains limited. One previous research by Hu et al. [18] indicates that microbial coexistence exhibits a stronger correlation with the resource niche dimensions, such as the gradient of soil organic matter, compared to the condition niche dimensions at local scales. In contrast, at a broader spatial scale (e.g., national or global scales), microbial coexistence may exhibit distinct variations along the condition niche dimensions, for example, across the soil pH gradient [14, 19]. Therefore, it could be predicted that microbial coexistence within forest soil exhibits varied responses to different ecological niche dimensions, and these responses are scale dependent. However, the explicit empirical examination of these predictions is still lacking. Herein, we aim to fill this knowledge gap by comparing the niche separation in microbial MEN properties and the MEN microbial diversity and exploring the mechanism of ecological niche that influences microbial occurrence relationships in forest soils. For simplicity, as cited in Yuan et al. [4], the term “biotic interactions” is employed to denote MEN properties, and “network community diversity” is utilized to unveil the occurrence and abundance characteristics of the network node.

In this study, natural forest soil samples were selected in China to investigate the niche separation of the biotic interactions and MEN community. Building upon previous research on the composition, diversity, and environmental response of forest soil microbial communities [14], we hypothesize the following: (i) considering the utilization strategies of microbial communities for dissolved organic matter (DOM) in forest soil will be influenced by its chemical characteristics [20], the chemical properties of forest soil DOM are likely the primary drivers of microbial biotic interactions and the structure of the MEN community, and (ii) given that the soil DOM is the most easily utilized source of carbon by microorganisms [15], the functional potential of microbial carbon metabolism in forest soils may be more closely associated with abiotic resource characteristics, compared to microbial ecological traits and soil variables.

## Material and Methods

### Soil Sampling and Soil Environmental Characteristics

Sixty-seven natural forest sample plots were selected within a 200 × 200 km range based on the forest distribution in China [21] using a simple random sampling method. The sampling area covers approximately 4000 km and includes various forest soil types and climatic zones in China. The climate characteristics, such as mean annual temperature (MAT) and mean annual precipitation (MAP), were obtained using data from the Qinghai-Tibet Plateau National Data Center [22]. The MAT of the sample plots ranges from − 1.3 to 23.7 °C, and the MAP ranges from 36 to 1921 mm (Table S1). In each forest sampling area, 8 to 12 sampling points were selected. After removing the surface litter layer at each sampling point, at least eight soil core samples with a diameter of 5 cm were collected. The topsoil (0–5 cm) was collected and transported to the laboratory on ice packs. In the laboratory, we further removed any roots and larger organic debris from the soil samples manually. Samples were then stored in a refrigerator at − 80 °C before subsequent soil variables measurements and environmental DNA extraction. Soil variables, including soil pH, soil nitrate nitrogen (NO_3_^−^) and ammonium nitrogen (NH_4_^+^), soil total phosphorus (TP), soil organic carbon (SOC), and dissolved organic carbon (DOC), were measured using previously established methods [23]. The DOM characteristics, including specific ultraviolet absorbance at 254 nm (SUVA_254_), the slope ratio (S_*R*_) of the 275–295 nm band to that of the 350–400 nm band, and the ratio of the ultraviolet absorbance at 250 nm and 365 nm (E_2_/E_3_), were measured utilizing our previously methods [24]. The E_2_/E_3_ and S_*R*_ values represent the relative molecular weight of DOM, with higher values indicating lower relative molecular weight [25, 26]. SUVA_254_ was employed as an indicator of soil DOM aromaticity,the higher the SUVA_254_ value, the greater the degree of soil DOM aromaticity [27].

### Environmental DNA Extraction and 16S rRNA Gene Sequencing and Analysis

FastDNA SPIN Kit for soil (MP Biochemicals, Solon, OH, USA) was used to extract total environmental DNA from the soil samples. The 16S rRNA V5 region sequences (784F/880R primer pair) were amplified by PCR and sequenced to explore the characteristics of soil microbial communities [16, 28, 29]. Electrophoresis (1.5% agarose gel) was used to detect the PCR products, and PCR products between 400 and 500 bp were mixed at the same density ratio. The E.Z.N.A.® gel extraction kit (Omega, USA) was utilized to purify PCR amplification. The sequencing library was prepared using the NEXTflex™ Rapid DNA-Seq Kit according to the standard protocol and then sequenced on the Illumina MiSeq PE250 high-throughput sequencing platform at Magigene Biotechnology Co., Ltd., Guangdong, China. Amplicon sequencing data were analyzed online at Majorbio Cloud Platform (www.majorbio.com). In brief, FLASH software (version 1.2.11) was used to merge and filter paired-end reads. The UPARSE software package analyzed sequences with 97% similarity to the same OTUs. Then, the sintax algorithm in USEARCH matches the representative sequence against the SILVA database (release 132). The RDP classifier performs taxonomic analysis of OTU representative sequences at the 97% similarity. We used OTUs with 97% similarity rather than amplicon sequence variants, as the latter produced more taxonomic groups and the abundance matrix was sparser, which could lead to greater bias in estimating network construction correlation [4]. The raw sequences were deposited in the NCBI SRA database under BioProject PRJNA988938.

### MEN Construction, Characterization, and Differentiation

We retained OTUs with more than 10 sequences and resampled all samples to the same sequencing depth, based on the minimum sequence count (41,159 sequences, Table S2) used for the MEN network construction. The molecular ecological network analysis pipeline (MENAP, http://ieg2.ou.edu/MENA/) was used to construct a microbial ecological network. MENs correlation was established based on the Pearson correlation of OTU abundance after logarithmic transformation, and the correlation cutoff threshold (St) was automatically determined based on the random matrix theory method [2]. To ensure the reliability of correlation calculations, only OTUs present in more than half of the soil samples were included for analysis. After determining St for each correlation matrix, an adjacency matrix was generated, containing only significant interrelationships where the absolute value of the coefficients (correlation strength) is greater than or equal to St values.

The MENAP encompasses the analysis of network-level topology characteristics, node-level topology characteristics, and network module identification. The overall MEN’s network topology is characterized using various topological indexes at the network level, including the number of nodes and edges (links of node), average degree (avgK), average clustering coefficient (avgCC), geodesic distance (GD), degree centralization (CD), stress centrality centralization (CS), betweenness centralization (CB), connectedness (Con), transitivity (Trans), and modularity index. Additionally, individual node topological indexes such as node clustering coefficient (node CC), node degree, node stress, and node betweenness are used to characterize the topological properties of each network node. A comprehensive introduction and calculation method of these indices have been described in Deng et al. [2].

MEN are divided into global and local MEN based on the range of soil samples. Global MEN refers to a network incorporating a comprehensive sample set across all geographical locations and soil types (*n* = 67). Meanwhile, local MEN is characterized by a network confined to samples originating from a specific geographic region, encompassing a limited range of soil variables. For instance, when constructing a local MEN for acidic soils, soil samples with a pH value exceeding 6.5 were excluded to construct networks. Each MEN is independently built based on specific soil samples representing a continuous gradient of soil variables, enabling the assessment of network sizes (total number of nodes), complexity, and stability under different groups of soil variables. The complexity of MENs is revealed through their network-level and node-level topological properties as well as modularity characteristics. To evaluate robustness, random or targeted removal of network nodes is performed to calculate the average interaction strength among the remaining nodes. Additionally, we adopt the method proposed by Yang et al. [30] based on the relationship between the remaining node and network interaction strength.

### Statistical Analysis

The spatial distribution patterns of microorganisms were explored to reveal the dissimilarity of MEN communities. We classified the soil samples into two geographical groups based on the Qinling Mountains-Huaihe River Line (latitude ≈32°), an important geomorphologic, climatic, and soil boundary in China: the high-latitude regions (latitude ≥ 32°, *n* = 29) and the low-latitude regions (latitude < 32°, *n* = 38), to investigate the spatial heterogeneity of soil environmental variables, climate factors, and microbial communities. The “ggstatsplot” package in R, an extension of the “ggplot2” package for generating visual charts with comprehensive statistical test information, is utilized to compare differences in soil variables and climate factors among geographic groups [31]. The networks were visualized using Gephi 0.9, the node color was utilized to represent the OTU taxonomic or module classification, and node size was employed to depict the topological index of the OTU. Heat maps based on the “ComplexHeatmap” package in R [32] were used to display the relative abundance of network OTUs, their topological properties, and taxonomic classification. The node-level topological index differences of MEN between geographic groups were assessed using the Wilcoxon rank-sum test, a statistical method available in the “stats” package of R.

Bray–Curtis dissimilarity was utilized to evaluate the *β*-diversity of network communities. Based on the Bray–Curtis dissimilarity matrix, principal coordinate analysis (PCoA) and distance decay relationship (DDR) were employed to validate the spatial heterogeneity of network communities. Permutational multivariate analysis of variance (PERMANOVA) was conducted to assess the impact of different geographical groupings on the similarity of microbial communities. The DDR was calculated as the ordinary least-squares regression slope representing the relationship between geographic distance and network community dissimilarity. We calculated pairwise correlation coefficients to evaluate relationships among environmental factors. The canonical correspondence analysis (CCA) model (based on package “vegan” in R) was employed to investigate the impact of soil variables on the spatial heterogeneity of microbial communities. Additionally, random forest (RF) models were utilized to determine the contribution of soil variables to the network community dissimilarity. The PICRUSt2 was used to predict the potential carbon metabolic function of the soil microbial, explicitly focusing on carbon metabolism function information derived from the KEGG database [33]. The analysis was performed using default settings, where metabolic functions were inferred based on the taxonomic classification of 16S rRNA gene sequences. In brief, PICRUSt2 first normalized the copy number of 16S rRNA genes to estimate the abundance of functional genes, followed by the prediction of gene family abundances and the mapping of these abundances to metabolic pathways. Subsequently, we employed structural equation modeling (SEM) using the “lavaan” package in R to examine the relationships between climatic characteristics, soil variables, network community ecological characteristics (diversity and similarity), and carbon metabolism function. The diversity of the network communities was assessed based on the Shannon index, which was calculated as $H=-\sum \left(pi\times \mathrm{ln}\left(pi\right)\right)$, where *pi* represents the relative abundance of OTU. And similarity was determined by the average Bray–Curtis distance among OTUs.

All statistical analyses were performed in the R environment (v4.2.2; http://www.r-project.org/), using “vegan,” “stats,” “ggstatsplot,” “ComplexHeatmap,” “ggplot2,” “lavaan,” “randomForest,” and “gplots” packages.

## Results

### Microbial Biotic Interactions in China Forest Soils

Significant variations were found in soil pH, climatic factors (MAP and MAT), and soil DOM quantity (indicated by DOC) and chemical characteristics (SUVA_254_ and E_2_/E_3_) from high-latitude to low-latitude regions (*p* < 0.05) (Table S3, Figs. S1 and S2). Among them, the spatial variations of MAP and MAT were consistent, with lower temperatures and precipitation observed in the soil samples in the high-latitude than low-latitude regions. Conversely, the soil pH and SUVA_254_ of soil samples were significantly higher in high-latitude regions than in low-latitude regions (*p* < 0.001) (Fig. S2). There were no significant differences in SOC, TP, NO_3_^−^, NH_4_^+^, and S_*R*_ between high- and low-latitude groups (Table S3).

**Fig. 1:**
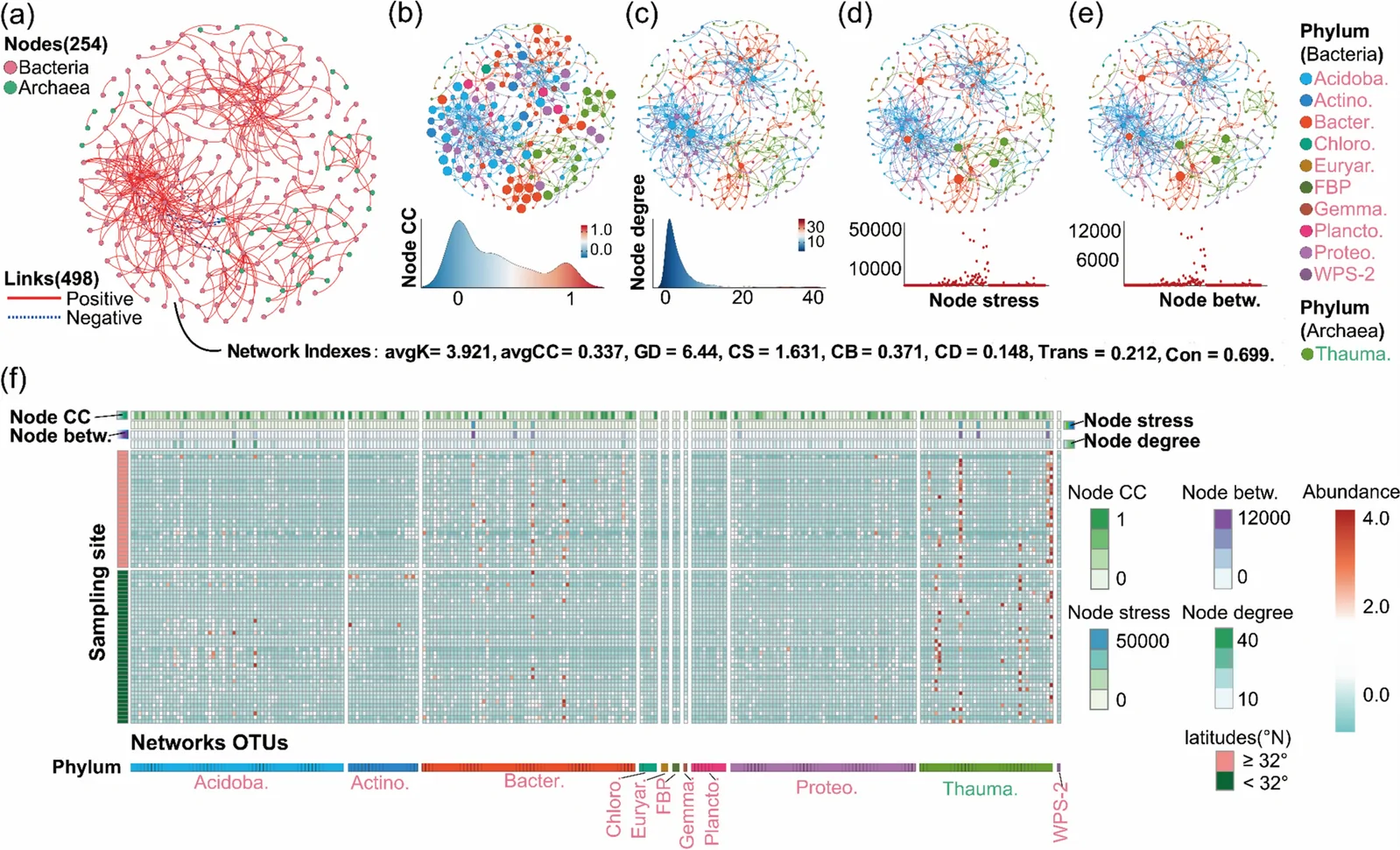
The global MENs across all sampling sites. **a** Visualization of constructed MEN of all soil samples. Nodes are colored to distinguish between bacterial phyla and archaea. The edge’s solid red and dashed blue lines (links) represent positive and negative correlations, respectively. The lower right corner indicates the network level topology index, avgK, average K; avgCC, average clustering coefficient; GD, geodesic distance; CS, centralization of stress centrality; CB, centralization of betweenness; CD, centralization of degree; Trans, transitivity; Con, connectedness. The size of each node is proportional to the **b** clustering coefficient (CC), **c** degree, **d** stress, and **e** betweenness of the OTUs. The diagram below shows the distribution of MEN node-level topological values. The color of the nodes represents the different microbial taxonomy in the phylum level. Features of MEN communities at the OTU level were visualized using heat maps (**f**). With sampling areas arranged along sampling latitude and divided into the high-latitude group (latitude > 32°, *n* = 29) and the low-latitude group (latitude < 32°, *n* = 38). Colors represent node-level features and node relative abundance. And the colorbar intensity represents the log-transformed abundance (log(reads + 1)) of the OTU. Node betw., node betweenness; Acidoba., Acidobacteria; Action., Actinobacteria; Bacter., Bacteroidetes; Chloro., Chloroflexi; Euryar., Euryarchaeota; Gemma., Gemmatimonadetes; Plancto., Planctomycetes; Proteo., Proteobacteria; Thauma., Thaumarchaeota

The global networks based on Pearson’s correlations of OTUs were constructed, followed by the best reference threshold (*St* = 0.78). The network size was 254 (total number of nodes). With 498 links, microbial species tended to co‐occur more (494 links were positive correlations) rather than co‐exclude (4 links were negative correlations) (Fig. 1a). Global networks exhibited a scale-free structure, indicated by the node degree distribution functions fitting well to power-law functions (*R*^2^ of 0.84–0.98). Additionally, the modular analysis result suggests that microbial OTUs in the network tend to form clusters and display modular structures (modularity index > 0.44). The node-level topological features, including the node CC, node stress, node betweenness, and node degree, are depicted in Fig. 1b, c, d, and e. In 254 nodes, approximately 30% of the nodes exhibited clustering coefficient values greater than 0.5 (values closer to 1 indicate the robust level of connectivity among network nodes) (Fig. 1b). Approximately, 80% of the nodes exhibited the node degree ranging from 0 to 5 (Fig. 1c), and the network “broker,” characterized by high stress and betweenness values, displayed a uniform distribution pattern (Fig. 1d, e). Overall, the results of node properties reveal that the network exhibits an elevated level of interaction but relies on specific connector OTUs. The taxonomic results show that Bacteroidetes, Acidobacteria, and Thaumarchaeota predominantly represented the OTUs with the highest stress and betweenness values in the network. Conversely, nodes with a high degree of connectivity (node degree) were primarily concentrated within the Acidobacteria, Bacteroidetes, and Proteobacteria phyla (Fig. 1f).

### Biogeographic Patterns of Global MEN Communities and Links with Soil Variations

**Fig. 2:**
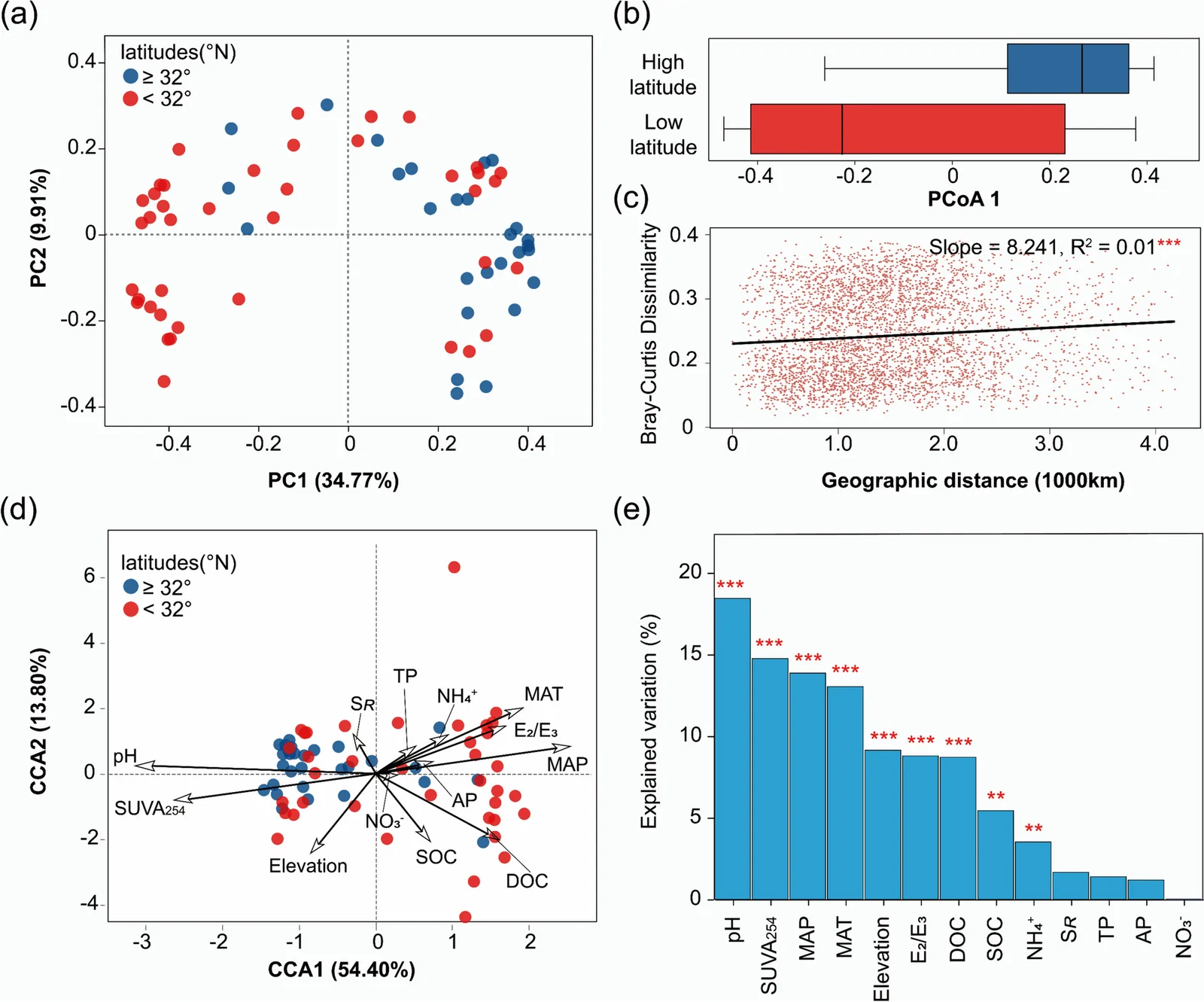
Spatial variations and influencing environmental factors of MEN community in OTU levels. **a** Scatter plot (1st and 2nd axis) of principal coordinate analysis (PCoA). Blue and red solid dots represent soil samples from high-latitude and low-latitude regions, respectively. **b** Community dissimilarities between high-latitude and low-latitude regions, based on the distribution of sample sites on 1st axis in PCoA. **c** The distance-decay curves illustrate the relationship between Bray–Curtis dissimilarity and geographic distances among sampling sites. **d** Canonical correlation analysis (CCA) was conducted to show the influence of soil variations in governing the biogeographic distribution of MEN communities and **e** identify the major soil environmental predictors based on CCA results based on total explained variation. Asterisks denote significant correlation (**p < 0.01; ***p < 0.001)

In the global MEN communities, significant spatial differences were observed in the relative abundance of dominant phyla from high-latitude to low-latitude regions. Specifically, a notable decrease in the relative abundance of Bacteroidetes was observed, while there was a substantial increase in the relative abundances of Acidobacteria, Proteobacteria, and Actinobacteria (Fig. S3). As indicated by the Bray–Curtis dissimilarity metrics in OTU levels, a significant DDR pattern spanning a geographic distance of ~ 4000 km, and MEN communities, tended to deviate from high-latitude to low-latitude regions (PERMANOVA, *R*^2^ = 0.134, *p* < 0.001) (Fig. 2a, b, and c). Furthermore, the CCA results showed a significant correlation between the dissimilarity of the microbial community and soil variations, including soil pH, climatic conditions (MAP and MAT), elevation in the sampling site, DOC, and soil DOM characteristics (SUVA_254_ and E_2_/E_3_) (Fig. 2d). Among them, pH, MAP, and SUVA_254_ were important soil variables that significantly influenced the similarity in MEN communities, accounting for 18.44%, 14.75%, and 13.86% of the total variance, respectively (Fig. 2e). Besides, soil pH, SUVA_254_, MAP, and DOC were important predictors for OTU dissimilarities of MENs based on RF analysis (Fig. S4). Additionally, we observed significant associations between pH, MAP, and SUVA_254_, with Pearson correlation coefficients exceeding 0.700 (Table S4).

### Changes in Microbial Interactions Across Forest Soil Niches

The CCA and RF model results show that abiotic conditions niche and abiotic resource niche types were respectively represented by soil pH and SUVA_254_, which were the first two important soil variables that influenced the MEN communities of forest soil microorganisms in this study (Figs. 2e and S4). To assess how microbial community biotic interactions change across forest soil niches, we compared the MEN networks across the pH and SUVA_254_ gradients.

**Fig. 3:**
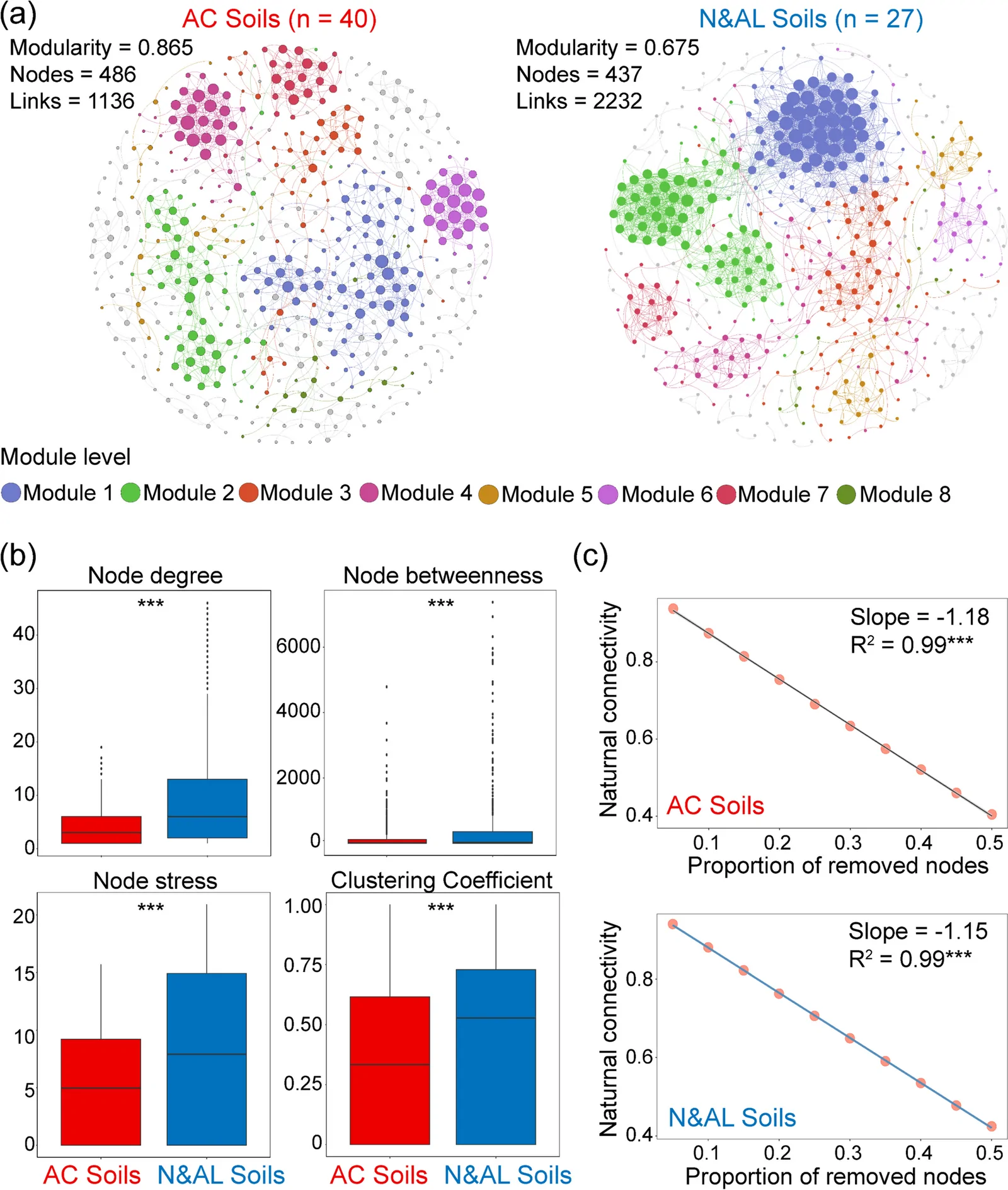
Microbial networks in acidic and non-acidic forest soils. **a** Overall dynamics of microbial co-occurrence networks between acidic soils (*n* = 40) and non-acidic soils (*n* = 27). Network nodes were colored by modularity. Network modularity, size (node numbers), and connectivity (link numbers) are shown for each network. Details of network topological properties are listed in Table S5. **b** Comparison of the unique node-level topological features, including node degree, betweenness, stress, and closeness centrality between acidic and non-acidic forest soils (***p < 0.001; Wilcoxon rank-sum test). **c** The robustness of microbial networks. A decreasing trend in natural connectivity is fitted with a 50% loss of nodes, and the diagrams display the *R*.^2^ value and slope. A lower absolute value of the slope indicates a more stable network. *n* represents the number of sample sites. Asterisks indicate a significant correlation (***p < 0.001)

The soil conditions niche type was divided into acidic (pH < 6.5, *n* = 40) and non-acidic (pH ≥ 6.5, *n* = 27) soils based on soil pH properties. MEN analysis showed that the acidic and non-acidic soil microbial communities network was constructed with a correlation threshold (*St* = 0.79), such that networks had 486 and 437 nodes with 1136 and 2232 links, respectively (Fig. 3a). Compared to the acidic soil, the non-acidic soil microbial MEN exhibited higher avgK, avgCC, and GD properties and increased Con and Trans. Additionally, the CB, CD, and CS in microbial MEN demonstrated greater values in non-acidic soil than in acidic soil, and lower modularity indexes were observed in non-acidic soil (0.675) compared to acidic soil (0.865) microbial MEN (Table S5). Our findings demonstrated that the MEN communities in non-acidic soils were more complex than those in acidic soils, and an increase in soil pH led to a reduction in the modularity of MEN. Furthermore, the values of network indexes for individual nodes, such as node degree, node stress, node betweenness, and node CC, were significantly higher in non-acidic soil MENs than in the acidic soils MENs, revealing a closer relationship among non-acidic soil microbial MENs compared to acidic soil (Fig. 3b). Based on the relationship between the interaction strength and the number of the remaining nodes, our result indicated that the networks showed slightly more stable relationship in non-acidic soil than in acidic soil, with slopes of − 1.15 and − 1.18, respectively (Fig. 3c).

According to the soil DOM aromaticity revealed by SUVA_254_ values, soil resource niche types were classified into high-aromaticity soils (SUVA_254_ > 2.00, *n* = 36) and low-aromaticity soils (SUVA_254_ < 2.00, *n* = 31). The microbial MEN (*St* = 0.79) comprised 466 and 512 nodes with 1136 and 2232 links for high- and low-aromaticity soils, respectively. Comparing the MEN network properties in between high- and low-aromaticity soils, it was observed that the high-aromaticity soil microbial community had higher avgK and CD but with lower avgCC, GD, CS, CB, Con, and Trans values (Table S5). This finding reveals a nonuniform fluctuation along the soil resources niche axis.

### Potential Effects of MEN Communities and Soil Variations on Microbial Carbon Metabolism

**Fig. 4:**
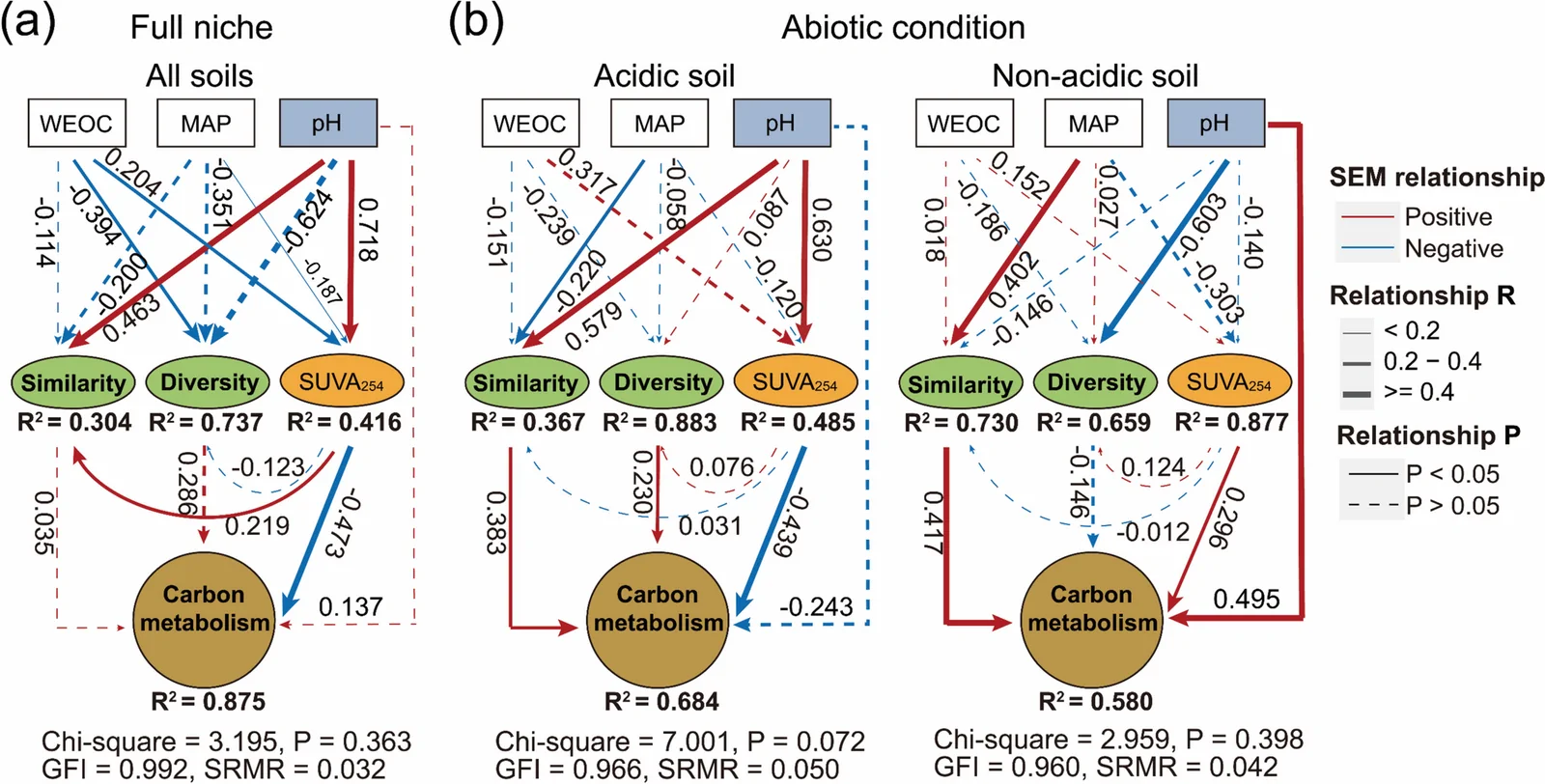
SEM models illustrating the effects of direct and indirect variables on microbial carbon metabolism. We examined the effects of MAP, soil pH, SUVA_254_, and the MEN community’s diversity and similarity on soil carbon metabolic potential at full niche scales (**a**) and between soil conditions niche (**b**), respectively. The goodness of fit of the model is shown at the bottom of this figure. GFI, goodness-of-fit indices; SRMR, standardized root-mean-square residual. Arrow widths are proportional to the strength of the relationship, the solid lines indicate significant associations (*p* < 0.05), and the numbers next to the arrow lines show the path coefficients

SEM was employed to investigate the direct and indirect effects of soil conditions, soil resources, and the ecological characteristics (diversity and similarity) of MEN communities on the functional potential of carbon metabolism in soil microorganisms. Considering the relatively significant impacts of MAP and DOC on the spatial heterogeneity of MEN communities (Fig. S4), these two variables are also incorporated into the SEM analysis as influential factors in the ecological characteristics of MEN communities. In the full niche scale (model containing all soil samples), the microbial carbon metabolism functional potential was directly and negatively associated with soil SUVA_254_ characters, and soil pH and DOC indirectly influenced microbial carbon metabolism via reductions in SUVA_254_ (Fig. 4a). Then, we compared the direct and indirect effects of MEN community ecological characteristics and soil variables on the potential of microbial carbon metabolism between the two soil pH groups. The functional potential of carbon metabolism in acidic soil was positively regulated by the diversity and similarity of MEN communities. In contrast, the soil SUVA_254_ exerted a negative regulatory effect. In non-acidic soil, the carbon metabolism was significantly positively regulated by the SUVA_254_ values, pH, and MEN communities’ similarity (Fig. 4b).

## Discussion

Forest ecosystems harbor a substantial portion of the biodiversity of terrestrial microbiomes on Earth [34]. The influence of microbial biotic interactions and abiotic factors on the microbial biogeographic pattern has been well-studied in forest ecosystems [19, 35]. However, to date, insufficient information is available on the dynamic association of biotic interactions with abiotic factors in forest soil. This study found that soil pH was the most important in determining the spatial variations of MEN communities in China forest soils, followed by DOM aromaticity (SUVA_254_). However, the functional potential of carbon metabolism in soil microorganisms is predominantly constrained by soil SUVA_254_ while weakly related to the soil pH.

Consistent with the previous report [36], our study reinforces the importance of soil pH as the prominent soil condition influencing the biotic interactions in Chinese forest soil microbial communities, which aligns with our first hypothesis. Soil pH significantly influences microbial ecology, and even a relatively small change in pH can have profound effects on the abundance and co-occurrence patterns of soil microbial species [37]. Typically, local soil microbial communities can be selected for in both acidic and alkaline soils, with “acidic specialists” predominating in acidic soils and “alkaline specialists” in alkaline soils [38]. The present study reveals that high soil pH enhances the biotic interactions among forest soil microbiomes. In comparison to acidic soils (soil pH ≤ 6.5), non-acidic soils (soil pH > 6.5) exhibit significantly heightened microbial network complexity, thereby highlighting that biotic interactions were apparent differentiation in the niche types of abiotic conditions.

DOM represents the most labile and biologically available fraction of soil organic carbon. Being the primary soluble carbon source for soil microorganisms, DOM significantly influences microbial abundance, community composition, diversity, and metabolic activities, including carbon cycling [39–41]. The chemical characteristics of DOM, such as its aromaticity degree, are determined by the bioavailability and reactivity of DOM, which are closely associated with soil microbial communities [3, 7, 18, 40, 41] . For example, the promotion of microbial diversity can be facilitated by increasing the diversity of DOM [42]. Therefore, high soil aromaticity might promote the coexistence of microbiota in complex soil environments. Our study demonstrates a significant correlation between DOM aromaticity and the quality of network interactions, indicating the importance of DOM characteristics in shaping microbial biotic interactions. The MEN size generally exhibited a significant increase from low to high DOM aromaticity, while soils with high DOM aromaticity displayed higher complexity in the microbial network (Text S1 and Fig. S5). However, it is worth noting that no apparent complexity gradient was observed in microbial biotic interactions. For example, MEN of soil microorganisms in the medium-aromaticity region exhibited the most elevated avgK and the lowest modularity (Fig. S5, the case of the medium-aromaticity region may be attributed to heightened environmental stress, which is typically determined by soil conditions [10]. It suggests that variation in soil resources does not dominate the microbial biotic interactions. This highlights our first hypothesis that the key regulation of abiotic resources in microbial biotic interactions may be subjective or otherwise non-representative. Overall, biotic interactions of forest soil coexisting microbial communities are more apparent concerning the soil condition than resource niche axes.

To examine our second hypothesis, the impact of MEN microbial characteristics and soil variables on microbial carbon metabolism potential was investigated through SEM across all soil niches, aiming to elucidate the key biotic and abiotic responses of microbial carbon metabolism. However, the SEM analysis did not observe the anticipated association between carbon metabolic function potential and soil abiotic conditions. Notably, a significant correlation was found between observed soil DOM aromaticity and carbon metabolic function, suggesting that the soil DOM aromaticity exerted a predominant influence on microbial carbon metabolism. Furthermore, our findings demonstrated a significant negative regulation of microbial carbon metabolism in acidic soils by the SUVA_254_. In non-acidic soils with soil pH greater than 6.5, there was a positive correlation between microbial carbon metabolism and SUVA_254_. In terrestrial soil environments, higher aromatic compounds in soil DOM are associated with lower bioavailability to microbial communities, which may in turn influence the relative enrichment of functional genes related to microbial carbon metabolism [15, 43]. This phenomenon also accounts for the significant negative correlation observed between soil DOM aromaticity and microbial carbon metabolism potential across all soil samples and in acidic soil samples. However, in non-acidic soils, our findings demonstrated that the increased aromaticity of soil DOM conferred benefits to the enrichment of microbial carbon metabolism. This phenomenon may be due to the non-acidic soil environment being conducive to microbial DOM degradation function [44], or the heightened level of DOM aromaticity promotes the enhancement of functional diversity within microbial communities [45]. Such enhancement is often sustained by microbial communities’ extensive functional redundancy characteristics [46]. Collectively, our results suggest that soil pH significantly regulates the functional potential of microbial carbon metabolism at the local scale and influences the ecological characteristics of microbial community MEN. Therefore, we have refined the second hypothesis of this study, which posits that abiotic resources primarily influence the functional potential of carbon metabolism at the full scale. However, at the local scale, the functional potential of carbon metabolism within microbial communities is influenced by a complex interplay between biotic interactions and abiotic factors.

Our results align with the metabolic theory of microbial ecology [47]. Although we did not directly measure the nutritional niches, our results imply that variations in abiotic resource niches, such as DOM characteristics, may have a notable influence on microbial community functions. The differentiation of biotic interactions along the abiotic conditions niche surpasses that of abiotic resources, likely due to the strong environmental filtering effect of factors such as soil pH [19]. Additionally, local-scale variables like forest vegetation further shape microbial co-occurrence patterns, potentially restricting the role of DOM chemistry [34]. In summary, our study unveils critical insights into the intricate interplay between soil properties, microbial network complexity, and stability. Our main conclusions are as follows:

1. Soil pH and other abiotic conditions have a greater impact on the niche differentiation of microbial communities compared to abiotic resources.
2. The functional potential of microbial communities is more strongly associated with resource niche differentiation than with environmental conditions.
3. Furthermore, given the strong linkage between microbial community structure and ecosystem functions, microbial network characteristics are promising as key indicators for evaluating the functional potential of microorganisms under environmental constraints.

Looking ahead, the dynamic nature of microbial communities on temporal scales prompts consideration of temporal dynamics in future studies of microbial networks [48]. As rapid environmental changes, such as climate change and land-use shifts, threaten biodiversity and ecosystem function, preserving, maintaining, and enhancing microbial interactions emerge as crucial strategies to mitigate these negative effects. By bolstering the stability of ecosystem functions, the biological interactions uncovered in our study offer promise in navigating the challenges of a rapidly changing world, underscoring the urgent need to safeguard microbial communities as custodians of ecosystem health and resilience.

## Supplementary Information

Below is the link to the electronic supplementary material.

Supplementary file1 (DOCX 3507 KB)

## Data Availability

The datasets presented in this study can be found in online repositories. The names of the repository/repositories and accession number(s) can be found below: https://www.ncbi.nlm.nih.gov/bioproject/PRJNA988938.
