# Supplementary material for: Forest Soil pH and Dissolved Organic Matter Aromaticity Are Distinct Drivers for Soil Microbial Community and Carbon Metabolism Potential
Source: Microb Ecol. 2025 Jan 27;87(1):177. doi: 10.1007/s00248-025-02493-5 (PMC11772527; doi:10.1007/s00248-025-02493-5)
Supplement: Supplementary file 1 — Supplementary file1 (DOCX 3507 KB) [file 248_2025_2493_MOESM1_ESM.docx]

**Supporting Information**

***Of***

**Forest soil pH and dissolved organic matter aromaticity distinctly drive microbial biotic** **interaction and carbon metabolism potential**

Zongxiao Zhang^1,2^, Qiang Zhang^1,2,3^, Xue Guo^4^, Zhenzhong Zeng^1,2^, Yinghui Wang^1,2^, Peng Zhang^1,2^, Dengzhou Gao^5^, Guisen Deng^1,2^, Guodong Sun^1,2^, Yuanxi Yang^1,2^, Junjian Wang^1,2,*^

^1^*State Environmental Protection Key Laboratory of Integrated Surface Water-Groundwater Pollution Control, School of Environmental Science and Engineering, Southern University of Science and Technology, Shenzhen, Guangdong 518055, China*

^2^*Guangdong Provincial Key Laboratory of Soil and Groundwater Pollution Control, Southern University of Science and Technology, Shenzhen, Guangdong 518055, China*

^3^*School of Environment, Harbin Institute of Technology, Harbin, 150090, China*

^4^ *State Key Joint Laboratory of Environment Simulation and Pollution Control, School of Environment, Tsinghua University, Beijing 100089, China*

*^5^Key Laboratory of Humid Subtropical Eco-geographical Process of Ministry of Education, College of Geographical Sciences, Fujian Normal University, Fuzhou 350000, China*

***Corresponding Author:** *Junjian Wang (Mailing Address: 1088 Xueyuan Road, Xili, Nanshan, Shenzhen, Guangdong, 518055, China; Email: wangjj@sustech.edu.cn).*

**Text S1. The more clear molecular ecological network (MEN) was divided along the soil aromaticity gradient**

To further clear the variation in soil microbial MEN along the resource niche dimensions, soil samples were classified into four soil groups based on SUVA_254_ characteristics: group 1 (SUVA_254_ < 1.00, *n* = 15) represented lowest aromatic soils, group 2 (1.00 < SUVA_254_ < 2.00; *n* = 21) represented medium aromatic soils, group 3 represented medium higher aromatic soils (2.00 < SUVA_254_ < 2.50, *n* = 17), and group 4 represented extremely highest aromatic soils (SUVA_254_ > 2.50, *n* = 14). The four SUVA_254_ MENs were constructed with a correlation threshold of 0.850. The network size tended to increase as the soil SUVA_254_ increased (from soil group 1 to group 4). The total number of MEN nodes from groups 1 to 4 amounted to 366, 334, 378, and 478, and has 591, 932, 487, and 967 links, respectively (**Figure S5a**). By contrast, the MEN topological indexes did not exhibit a SUVA_254_ gradient, the network topological indexes in the highest SUVA_254_ groups (group 4, soil SUVA_254_ ranged from 2.53 to 3.58) exhibited the highest geodesic distance, centralization of stress centrality, and connectedness, whereas the centralization of betweenness and transitivity of MEN were highest in the group with lowest SUVA_254_ (group 1, soil SUVA_254_ ranged from 0.26 to 0.95) (**Figure S5b**). Additionally, based on the resistance to node loss of the network, the MENs had the highest robustness in group 2 soil, and the lowest occurred in group 1 (**Figure S5c**).


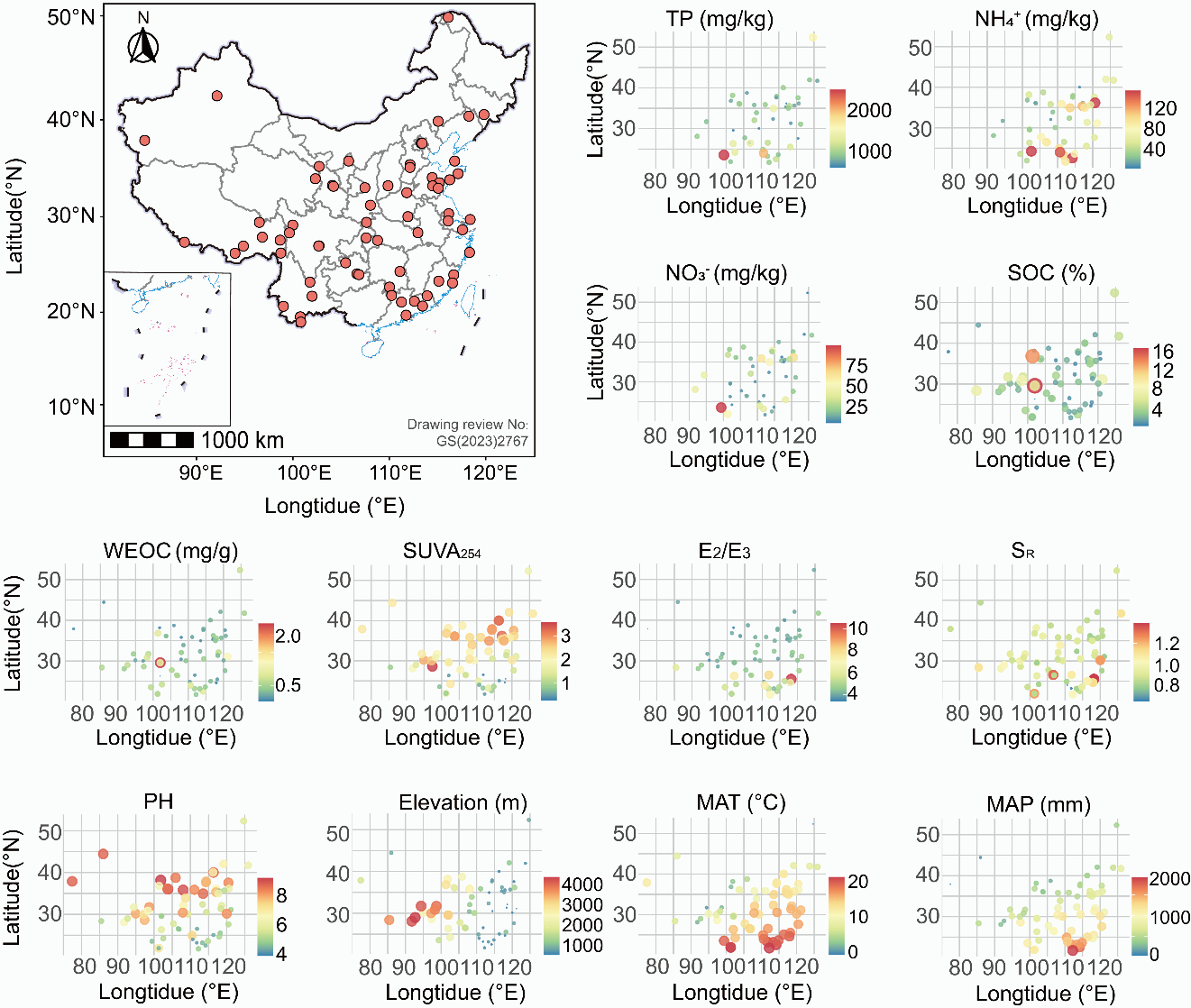
**Figure S1.** Sampling sites, spatial distribution of sampling site elevation, climatic characteristics, soil environmental properties, and DOM characteristics. The color and size of the scatter points represent soil variation values. MAT: mean annual temperature; MAP: mean annual precipitation; TP: total phosphorus; NH_4_^+^: ammonium nitrogen; NO_3_^-^: nitrate nitrogen; SOC: soil organic carbon; DOC: dissolved organic carbon; SUVA_254_: specific ultraviolet absorbance at 254 nm; E_2_/E_3_: the ratio of the fluorescence signal at 250 nm and 365 nm; S_R_: slope ratio of the 275–295 nm band to that of the 350–400 nm band.


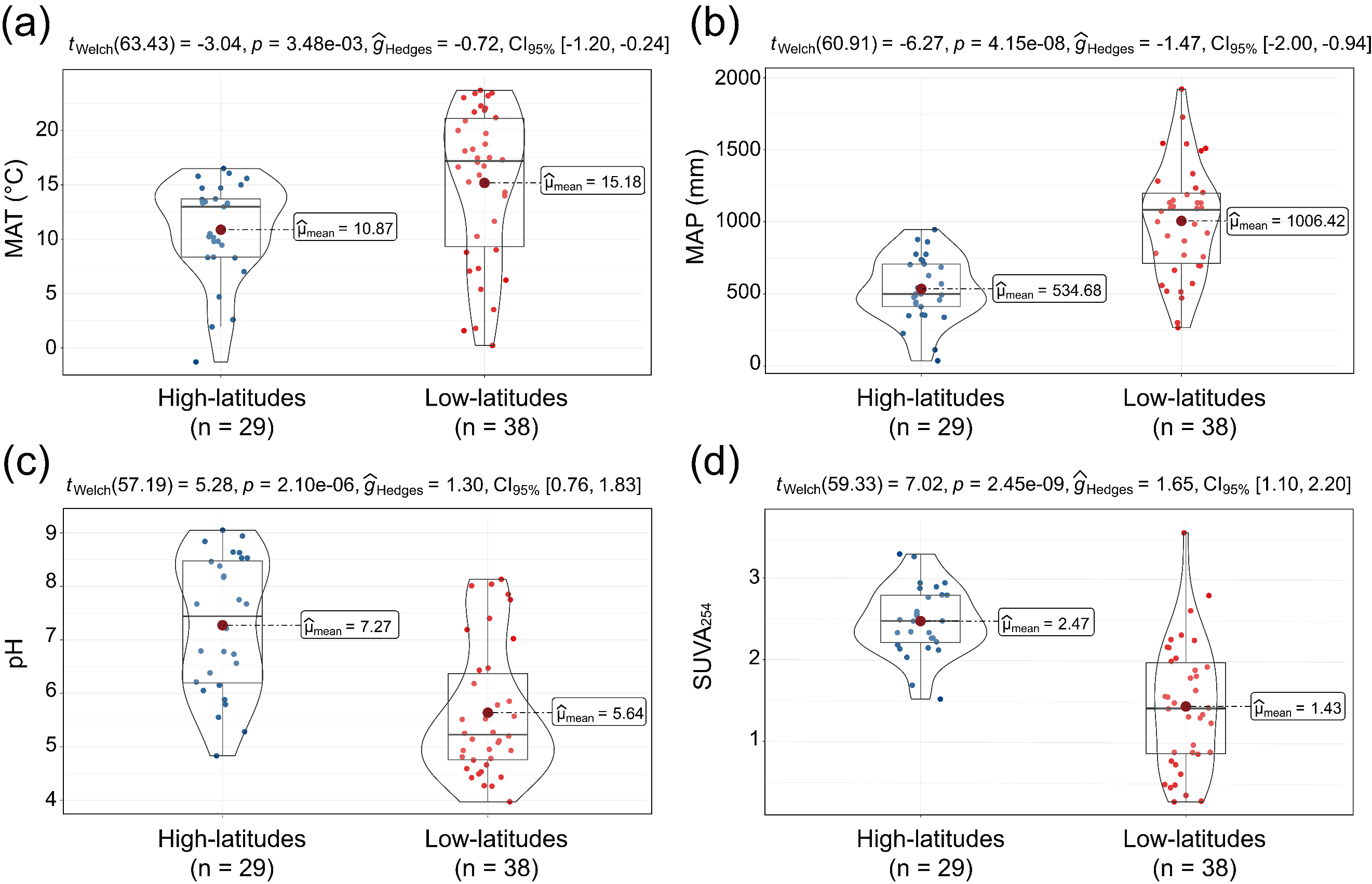


**Figure S2.** Comparison of MAT (**a**), MAP (**b**), soil pH (**c**), and SUVA_254_ (**d**) between high- and low-latitude regions based on Welch’s t-test. The parameter, statistic, significance, effect size type, estimate, confidence intervals, and mean value were reported in the plots.


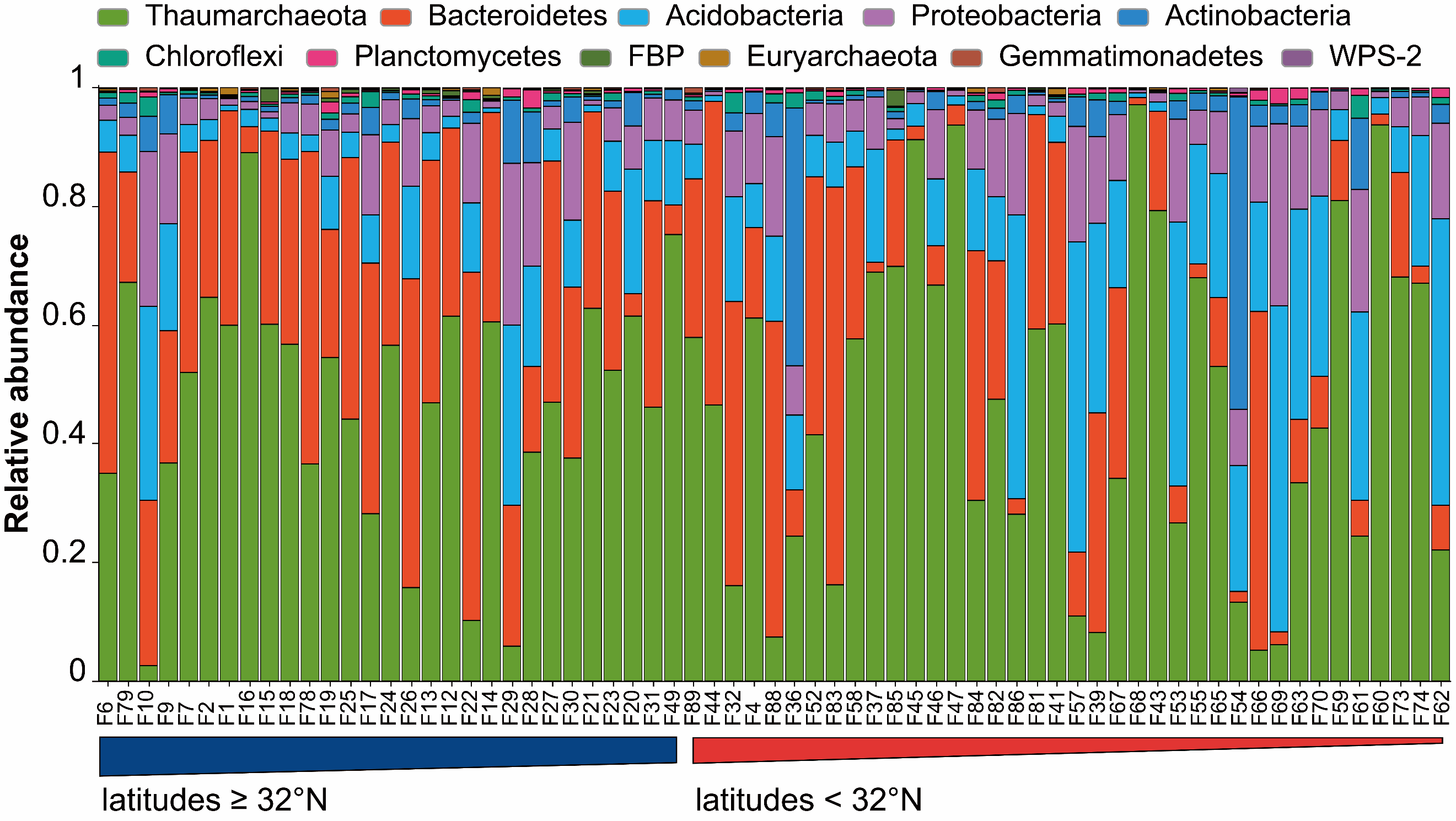


**Figure S3.** Taxonomic composition of MEN communities in phylum level. F1-F62 represents soil samples from high- to low-latitude regions. Blue and red represent soil samples from high latitudes and low latitudes regions, respectively.


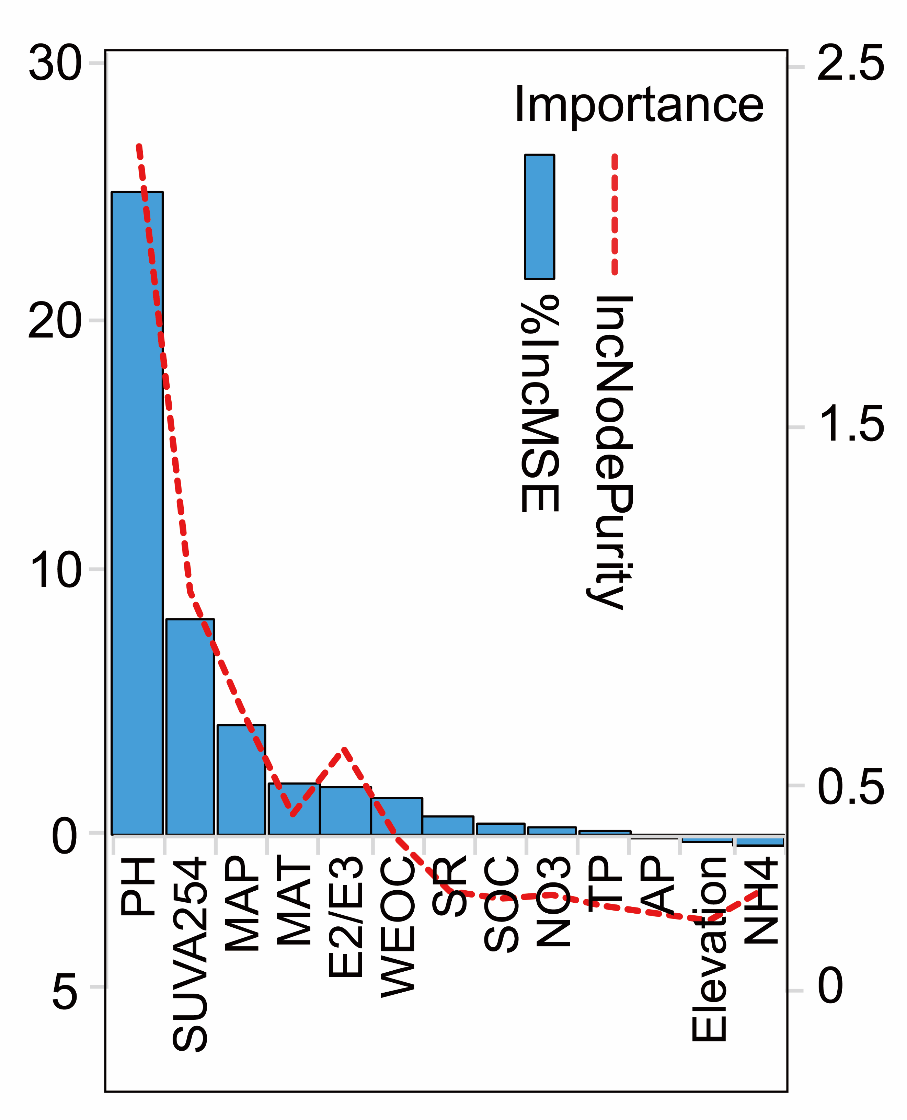
**Figure S4.** Importance ranking of soil variables affecting community similarity in MENs communities, based on random forest analysis. %IncMSE: Increase in mean squared error; IncNodePurity: Increase in node purity. SUVA254: SUVA_254_; E2/E3: E_2_/E_3_; SR: S*_R_*; NH4: NH_4_^+^; NO3: NO_3_^−^.


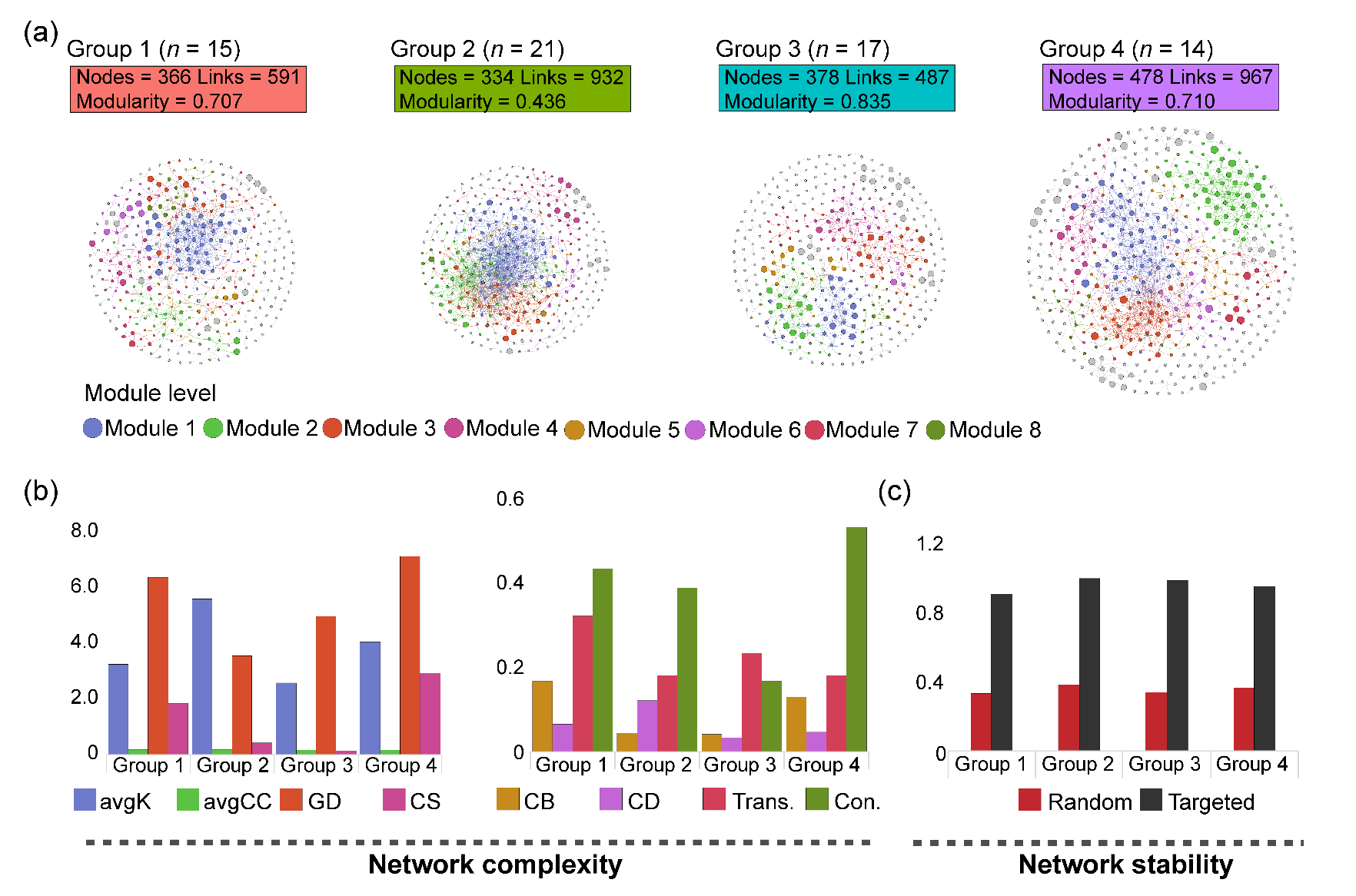
**Figure S5.** Overall dynamics of microbial local MENs between four soil aromaticity groups **(a)**, the network-level topological features **(b)**, and the robustness of microbial networks of four soil groups based on SUVA_254_ characteristics. *n* represents the number of sample sites. The size of each network is proportional to the node numbers. Network nodes were colored by modularity. Network modularity, size (node numbers), and connectivity (link numbers) are shown for each network. avgK: average K; avgCC: average clustering coefficient; GD: geodesic distance; CS: centralization of stress centrality; CB: centralization of betweenness; CD: centralization of degree; Trans: transitivity; Con: connectedness.

**Table S1.** The sample ID, site locations, climatic variables, soil proprieties and soil types in this study area

| Sample ID | Latitude  (°N) | Longtidue  (°E) | Elevation (m) | MAT  (°C) | MAP  (mm) | PH | TP  (mg/kg) | NH_4_^+^  (mg/kg) | NO_3_^-^  (mg/kg) | SOC  (%) | DOC (mg/g) | SUVA_254_ | E_2/_E_3_­­ | S*_R_* | Soil Type | Groups |
| --- | --- | --- | --- | --- | --- | --- | --- | --- | --- | --- | --- | --- | --- | --- | --- | --- |
| F1 | 40.010 | 116.390 | 45.000 | 13.442 | 441.472 | 8.530 | *NA* | *NA* | *NA* | 2.226 | 0.248 | 2.791 | 3.804 | 0.810 | Entisols | High-latitude |
| F10 | 42.072 | 119.163 | 685.000 | 8.292 | 354.917 | 6.780 | *NA* | *NA* | *NA* | 1.530 | 0.260 | 2.329 | 4.008 | 0.848 | Inceptisols | High-latitude |
| F12 | 36.036 | 103.820 | 1611.000 | 9.786 | 348.389 | 8.940 | 740.968 | 28.364 | 0.159 | 0.689 | 0.101 | 2.589 | 3.718 | 0.828 | Entisols | High-latitude |
| F13 | 36.134 | 103.688 | 1637.000 | 9.814 | 337.583 | 8.460 | 651.563 | 22.662 | 17.593 | 3.302 | 0.353 | 2.891 | 3.988 | 0.955 | Entisols | High-latitude |
| F14 | 35.817 | 108.011 | 2390.000 | 10.239 | 499.139 | 8.840 | 495.396 | 54.250 | 12.320 | 0.772 | 0.117 | 2.474 | 4.121 | 0.872 | Inceptisols | High-latitude |
| F15 | 38.162 | 101.844 | 2390.000 | 2.586 | 352.278 | 9.050 | 664.340 | 60.230 | 18.650 | 0.842 | 0.105 | 2.131 | 3.549 | 0.979 | Aridisols | High-latitude |
| F16 | 38.747 | 105.917 | 2009.000 | 4.694 | 225.611 | 8.380 | 368.210 | 47.220 | 19.520 | 1.437 | 0.152 | 2.143 | 4.026 | 0.851 | Entisols | High-latitude |
| F17 | 36.823 | 101.368 | 2707.000 | 1.925 | 489.000 | 6.380 | 424.495 | 31.486 | 29.452 | 13.324 | 0.853 | 2.537 | 4.005 | 0.890 | Entisols | High-latitude |
| F18 | 37.910 | 114.347 | 275.000 | 13.397 | 458.028 | 7.670 | 570.726 | 37.799 | 46.325 | 2.663 | 0.296 | 2.939 | 4.087 | 0.899 | Entisols | High-latitude |
| F19 | 37.608 | 114.286 | 273.000 | 13.658 | 476.750 | 7.750 | 365.123 | 40.240 | 22.250 | 1.595 | 0.209 | 2.873 | 4.116 | 0.928 | Entisols | High-latitude |
| F2 | 40.032 | 116.256 | 138.000 | 13.317 | 431.861 | 6.790 | 676.665 | 14.490 | 12.365 | 3.111 | 0.317 | 3.292 | 3.867 | 0.847 | Inceptisols | High-latitude |
| F20 | 32.363 | 113.366 | 350.000 | 15.800 | 861.278 | 5.550 | 341.475 | 41.332 | 5.315 | 0.877 | 0.278 | 2.180 | 4.487 | 0.794 | Inceptisols | High-latitude |
| F21 | 34.948 | 113.516 | 105.000 | 15.594 | 492.056 | 8.530 | 1334.411 | 111.409 | 54.216 | 0.844 | 0.103 | 2.939 | 4.161 | 0.903 | Inceptisols | High-latitude |
| F22 | 35.871 | 111.114 | 717.000 | 10.153 | 500.167 | 8.170 | 653.236 | 95.560 | 65.250 | 1.959 | 0.228 | 2.472 | 4.174 | 0.872 | Inceptisols | High-latitude |
| F23 | 33.900 | 108.643 | 927.000 | 9.458 | 626.889 | 7.210 | 575.561 | 32.294 | 6.898 | 1.667 | 0.257 | 2.557 | 4.347 | 1.051 | Inceptisols | High-latitude |
| F24 | 36.212 | 117.120 | 304.000 | 14.700 | 706.889 | 6.560 | 190.858 | 22.774 | 3.590 | 2.249 | 0.416 | 3.260 | 4.110 | 0.795 | Alfisols | High-latitude |
| F25 | 37.569 | 120.477 | 127.000 | 12.986 | 542.444 | 8.190 | 626.360 | 65.380 | 16.250 | 1.208 | 0.163 | 2.763 | 4.066 | 0.846 | Inceptisols | High-latitude |
| F26 | 36.173 | 120.641 | 874.000 | 10.511 | 738.250 | 6.050 | 418.694 | 158.802 | 67.799 | 0.798 | 0.258 | 2.487 | 4.303 | 0.835 | Inceptisols | High-latitude |
| F27 | 35.323 | 117.031 | 217.000 | 15.008 | 685.556 | 7.670 | 292.494 | 120.324 | 14.335 | 1.040 | 0.136 | 2.341 | 3.785 | 0.825 | Alfisols | High-latitude |
| F28 | 35.556 | 117.973 | 215.000 | 13.706 | 731.250 | 4.830 | 830.061 | 11.415 | 9.554 | 3.935 | 0.481 | 2.330 | 4.027 | 0.796 | Entisols | High-latitude |
| F29 | 35.681 | 119.396 | 300.000 | 13.175 | 775.500 | 5.280 | 376.559 | 65.461 | 28.715 | 2.214 | 0.315 | 2.117 | 4.198 | 0.832 | Alfisols | High-latitude |
| F30 | 34.984 | 117.716 | 297.000 | 14.706 | 702.917 | 6.210 | 620.994 | 99.440 | 2.620 | 3.291 | 0.397 | 2.788 | 4.127 | 0.904 | Entisols | High-latitude |
| F31 | 32.103 | 118.599 | 10.000 | 16.506 | 945.722 | 5.880 | *NA* | *NA* | *NA* | 2.878 | 0.643 | 1.517 | 4.574 | 0.914 | Inceptisols | High-latitude |
| F32 | 31.351 | 118.403 | 29.000 | 17.475 | 1074.222 | 5.520 | 336.254 | 69.360 | 5.360 | 1.326 | 0.341 | 1.221 | 4.413 | 0.888 | Inceptisols | Low-latitude |
| F36 | 30.526 | 114.437 | 28.000 | 18.122 | 1106.750 | 5.510 | *NA* | *NA* | *NA* | 2.573 | 0.468 | 1.299 | 3.928 | 0.938 | Inceptisols | Low-latitude |
| F37 | 30.064 | 109.332 | 1000.000 | 14.003 | 1203.528 | 5.780 | 224.638 | 37.777 | 4.619 | 2.774 | 0.374 | 1.869 | 4.327 | 0.913 | Ultisols | Low-latitude |
| F39 | 26.540 | 111.839 | 118.000 | 18.756 | 1131.250 | 4.430 | 882.743 | 50.582 | 5.830 | 0.913 | 0.168 | 0.339 | 8.117 | 1.074 | Ultisols | Low-latitude |
| F4 | 31.095 | 121.198 | 4.000 | 17.089 | 867.333 | 4.930 | 627.219 | 87.774 | 13.985 | 7.070 | 1.185 | 1.469 | 4.791 | 0.784 | Inceptisols | Low-latitude |
| F41 | 27.758 | 105.315 | 1508.000 | 14.317 | 922.694 | 5.620 | 552.262 | 90.483 | 6.050 | 2.946 | 0.543 | 2.016 | 4.544 | 0.866 | Ultisols | Low-latitude |
| F43 | 26.324 | 102.005 | 1944.000 | 16.739 | 695.472 | 5.850 | 1170.213 | 56.174 | 4.989 | 0.240 | 0.029 | 0.471 | 7.564 | 0.948 | Ultisols | Low-latitude |
| F44 | 31.722 | 98.664 | 3847.000 | 1.792 | 518.583 | 8.040 | *NA* | *NA* | *NA* | 5.204 | 0.617 | 1.978 | 4.150 | 0.968 | Inceptisols | Low-latitude |
| F45 | 29.597 | 102.049 | 2320.000 | 9.019 | 781.833 | 5.140 | *NA* | *NA* | *NA* | 5.993 | 0.996 | 1.541 | 4.962 | 0.979 | Inceptisols | Low-latitude |
| F46 | 29.586 | 102.026 | 2778.000 | 7.303 | 769.528 | 3.970 | 235.188 | 48.120 | 10.700 | 16.252 | 2.354 | 1.398 | 4.520 | 0.797 | Inceptisols | Low-latitude |
| F47 | 29.574 | 101.997 | 3003.000 | 5.389 | 757.667 | 4.950 | 181.506 | 37.188 | 5.993 | 6.400 | 0.926 | 2.152 | 4.332 | 0.796 | Inceptisols | Low-latitude |
| F49 | 32.073 | 108.040 | 720.000 | 16.069 | 877.389 | 6.730 | 769.995 | 15.949 | 29.883 | 3.676 | 0.422 | 2.028 | 4.550 | 0.930 | Inceptisols | High-latitude |
| F52 | 30.401 | 107.922 | 542.000 | 17.511 | 1095.111 | 8.010 | *NA* | *NA* | *NA* | 1.446 | 0.160 | 2.300 | 3.955 | 0.873 | Inceptisols | Low-latitude |
| F53 | 25.503 | 118.226 | 588.000 | 20.008 | 1150.000 | 4.780 | 1035.657 | 61.103 | 42.624 | 0.912 | 0.181 | 0.260 | 10.519 | 1.393 | Ultisols | Low-latitude |
| F54 | 24.645 | 117.968 | 79.000 | 21.883 | 903.056 | 5.080 | *NA* | *NA* | *NA* | 0.575 | 0.100 | 0.269 | 6.372 | 1.128 | Ultisols | Low-latitude |
| F55 | 25.049 | 116.365 | 207.000 | 21.183 | 1235.056 | 4.420 | *NA* | *NA* | *NA* | 1.812 | 0.344 | 0.433 | 5.360 | 1.054 | Ultisols | Low-latitude |
| F57 | 27.616 | 120.481 | 316.000 | 18.281 | 1283.000 | 5.250 | 234.742 | 63.248 | 18.351 | 1.714 | 0.342 | 1.286 | 4.791 | 0.810 | Ultisols | Low-latitude |
| F58 | 30.157 | 120.031 | 52.000 | 17.311 | 1189.083 | 8.130 | *NA* | *NA* | *NA* | 2.318 | 0.244 | 2.245 | 4.454 | 1.238 | Ultisols | Low-latitude |
| F59 | 23.213 | 111.784 | 44.000 | 23.028 | 1541.889 | 4.270 | 631.250 | 110.896 | 40.843 | 1.589 | 0.478 | 0.759 | 5.399 | 0.664 | Ultisols | Low-latitude |
| F6 | 52.340 | 124.693 | 404.000 | -1.303 | 411.472 | 5.790 | 1571.466 | 66.705 | 1.929 | 4.827 | 0.681 | 1.687 | 4.034 | 0.890 | Inceptisols | High-latitude |
| F60 | 22.558 | 114.199 | 72.000 | 23.203 | 1726.972 | 4.490 | 927.225 | 158.360 | 9.990 | 3.979 | 0.842 | 0.863 | 4.916 | 0.853 | Ultisols | Low-latitude |
| F61 | 23.176 | 113.297 | 216.000 | 22.281 | 1510.333 | 4.590 | *NA* | *NA* | *NA* | 1.822 | 0.447 | 1.549 | 4.329 | 0.806 | Inceptisols | Low-latitude |
| F62 | 21.739 | 112.221 | 197.000 | 23.703 | 1921.222 | 4.930 | 631.742 | 99.600 | 5.400 | 1.372 | 0.395 | 0.465 | 7.193 | 1.029 | Ultisols | Low-latitude |
| F63 | 23.616 | 114.833 | 226.000 | 22.061 | 1545.333 | 5.270 | 544.129 | 82.440 | 4.120 | 1.863 | 0.422 | 0.954 | 5.065 | 0.943 | Inceptisols | Low-latitude |
| F65 | 24.930 | 110.485 | 347.000 | 19.736 | 1493.556 | 7.400 | 678.962 | 103.394 | 4.711 | 1.556 | 0.186 | 1.622 | 3.815 | 0.818 | Ultisols | Low-latitude |
| F66 | 24.004 | 110.676 | 170.000 | 20.906 | 1334.167 | 4.260 | 2112.325 | 151.767 | 38.259 | 2.788 | 0.799 | 0.597 | 7.424 | 0.650 | Ultisols | Low-latitude |
| F67 | 26.540 | 106.664 | 1256.000 | 15.908 | 983.889 | 4.810 | 294.221 | 82.893 | 6.131 | 2.195 | 0.372 | 0.843 | 4.109 | 1.318 | Ultisols | Low-latitude |
| F68 | 26.445 | 106.893 | 1212.000 | 15.278 | 1001.417 | 4.750 | 1261.341 | 98.267 | 11.093 | 3.179 | 0.565 | 1.327 | 4.940 | 0.834 | Entisols | Low-latitude |
| F69 | 24.239 | 102.535 | 1870.000 | 16.647 | 664.250 | 5.200 | 1173.162 | 158.655 | 2.520 | 1.067 | 0.670 | 2.234 | 5.105 | 0.812 | Ultisols | Low-latitude |
| F7 | 41.734 | 125.936 | NA | 7.017 | 775.000 | 6.150 | 408.971 | 66.317 | 16.817 | 4.690 | 0.486 | 2.265 | 4.709 | 1.106 | Alfisols | High-latitude |
| F70 | 23.551 | 99.376 | 1178.000 | 21.714 | 1096.222 | 4.660 | 2787.936 | 52.135 | 106.602 | 3.512 | 0.602 | 0.714 | 5.581 | 0.864 | Ultisols | Low-latitude |
| F73 | 21.910 | 101.270 | NA | 23.417 | 1132.778 | 7.020 | 566.370 | 44.660 | 56.230 | 3.856 | 0.498 | 1.796 | 4.888 | 1.232 | Ultisols | Low-latitude |
| F74 | 21.900 | 101.270 | NA | 23.458 | 1133.556 | 4.530 | *NA* | *NA* | *NA* | 2.660 | 0.598 | 0.865 | 5.183 | 0.840 | Ultisols | Low-latitude |
| F78 | 37.893 | 77.409 | 1343.000 | 13.339 | 35.917 | 8.640 | *NA* | *NA* | *NA* | 0.601 | 0.106 | 2.260 | 3.401 | 0.860 | Aridisols | High-latitude |
| F79 | 44.447 | 85.986 | 388.000 | 8.325 | 112.333 | 8.630 | *NA* | *NA* | *NA* | 1.478 | 0.137 | 2.219 | 4.011 | 0.840 | Aridisols | High-latitude |
| F81 | 28.103 | 91.771 | 4123.000 | 1.575 | 301.111 | 6.430 | 632.159 | 33.360 | 43.250 | 2.700 | 0.449 | 1.909 | 4.567 | 1.005 | Inceptisols | Low-latitude |
| F82 | 28.617 | 97.365 | 2139.000 | 11.653 | 1093.056 | 7.750 | *NA* | *NA* | *NA* | 2.145 | 0.522 | 3.582 | 3.983 | 0.892 | Entisols | Low-latitude |
| F83 | 30.168 | 94.937 | 2289.000 | 10.264 | 697.028 | 7.850 | *NA* | *NA* | *NA* | 2.234 | 0.228 | 2.784 | 3.651 | 0.869 | Entisols | Low-latitude |
| F84 | 28.986 | 92.694 | 4314.000 | 3.519 | 266.750 | 5.570 | *NA* | *NA* | *NA* | 3.542 | 0.638 | 1.407 | 4.603 | 1.027 | Inceptisols | Low-latitude |
| F85 | 30.004 | 97.181 | 3402.000 | 8.792 | 572.139 | 7.190 | *NA* | *NA* | *NA* | 2.260 | 0.168 | 2.597 | 4.144 | 0.976 | Gelisols | Low-latitude |
| F86 | 28.386 | 85.431 | 3539.000 | 7.069 | 513.944 | 5.110 | *NA* | *NA* | *NA* | 6.866 | 0.561 | 0.866 | 5.899 | 1.101 | Gelisols | Low-latitude |
| F88 | 30.862 | 98.283 | 3654.000 | 6.236 | 471.333 | 6.470 | *NA* | *NA* | *NA* | 7.489 | 0.756 | 1.773 | 4.416 | 0.898 | Inceptisols | Low-latitude |
| F89 | 31.722 | 94.394 | 4084.000 | 0.217 | 558.750 | 6.180 | 547.269 | 25.320 | 44.170 | 5.824 | 0.451 | 2.146 | 4.024 | 0.927 | Inceptisols | Low-latitude |
| F9 | 41.954 | 123.725 | 180.000 | 8.358 | 569.694 | NA | 567.500 | 73.240 | 4.160 | NA | NA | NA | NA | NA | Inceptisols | High-latitude |

*NA* indicates the missing value

**Table S2.** The sample ID and sequencing data information.

| Sample ID | Raw total reads | Clean total reads | Q20(%) | Q30(%) | GC(%) | Sequences number |
| --- | --- | --- | --- | --- | --- | --- |
| F1 | 89323 | 88438 | 99 | 96.1 | 51.9 | 64230 |
| F10 | 84533 | 83829 | 99 | 96.3 | 52.9 | 53338 |
| F12 | 91882 | 91218 | 98.9 | 96 | 54.9 | 50981 |
| F13 | 86946 | 86113 | 99.1 | 96.3 | 51.1 | 55488 |
| F14 | 88638 | 87953 | 99.1 | 96.5 | 51.8 | 47103 |
| F15 | 88571 | 87757 | 99 | 96.2 | 53.7 | 52456 |
| F16 | 86566 | 85829 | 99 | 96.4 | 54.1 | 49394 |
| F17 | 88775 | 87965 | 99 | 96.3 | 52.2 | 49119 |
| F18 | 85851 | 85096 | 99.1 | 96.4 | 52.4 | 43864 |
| F19 | 86733 | 85978 | 99 | 96.3 | 51.9 | 41940 |
| F2 | 85868 | 85090 | 98.9 | 96.1 | 52.9 | 58100 |
| F20 | 90000 | 89348 | 99 | 96.2 | 55.2 | 51306 |
| F21 | 89980 | 89208 | 99.1 | 96.4 | 52.7 | 50181 |
| F22 | 85925 | 85107 | 99 | 96.2 | 52.5 | 60107 |
| F23 | 84305 | 83354 | 98.9 | 96 | 53.8 | 50130 |
| F24 | 86954 | 86312 | 99 | 96.3 | 54.9 | 64763 |
| F25 | 88330 | 87535 | 99 | 96.2 | 51.9 | 42542 |
| F26 | 85621 | 84644 | 99 | 96.1 | 52.2 | 52894 |
| F27 | 87099 | 86178 | 99 | 96.2 | 53.6 | 43754 |
| F28 | 84448 | 83671 | 99 | 96.3 | 51.7 | 52504 |
| F29 | 91630 | 90717 | 99 | 96.2 | 52.3 | 56523 |
| F30 | 87969 | 87189 | 99.1 | 96.4 | 51.8 | 47198 |
| F31 | 84630 | 83691 | 98.9 | 96 | 52.1 | 49577 |
| F32 | 85208 | 84509 | 99 | 96.3 | 54 | 45987 |
| F36 | 90187 | 89334 | 99 | 96.2 | 53.4 | 53367 |
| F37 | 89692 | 88708 | 99 | 96.2 | 52.8 | 44205 |
| F39 | 87211 | 86216 | 99 | 96.1 | 53 | 52182 |
| F4 | 87737 | 86941 | 99 | 96.3 | 52.9 | 47887 |
| F41 | 87312 | 86642 | 98.9 | 96 | 56.2 | 41836 |
| F43 | 91430 | 90803 | 99 | 96.4 | 56.2 | 43682 |
| F44 | 90268 | 89406 | 99.1 | 96.4 | 52.8 | 49398 |
| F45 | 90294 | 89517 | 99 | 96.1 | 51.8 | 46157 |
| F46 | 87906 | 87304 | 98.9 | 95.9 | 53.6 | 49619 |
| F47 | 84559 | 83875 | 99 | 96.2 | 50.7 | 45961 |
| F49 | 91416 | 90742 | 98.8 | 95.6 | 55.3 | 45920 |
| F52 | 86253 | 85687 | 98.9 | 95.9 | 55.3 | 50852 |
| F53 | 84991 | 84292 | 98.8 | 95.7 | 54.3 | 59577 |
| F54 | 92101 | 91302 | 98.7 | 95.3 | 55.5 | 61437 |
| F55 | 86432 | 85800 | 98.9 | 96 | 52.7 | 64142 |
| F57 | 84694 | 83940 | 98.9 | 95.9 | 55.8 | 57484 |
| F58 | 84399 | 83924 | 99 | 96.2 | 55.3 | 63863 |
| F59 | 90632 | 90016 | 98.9 | 95.9 | 56.2 | 62724 |
| F6 | 89089 | 88392 | 99 | 96.1 | 54 | 53126 |
| F60 | 90116 | 89467 | 98.9 | 96 | 53 | 53669 |
| F61 | 84503 | 83846 | 98.9 | 95.9 | 55.7 | 46318 |
| F62 | 89123 | 88496 | 98.8 | 95.6 | 54.4 | 61083 |
| F63 | 88736 | 88078 | 99 | 96.4 | 55 | 64109 |
| F65 | 92194 | 91416 | 98.9 | 95.9 | 54.9 | 52561 |
| F66 | 88556 | 87855 | 98.9 | 96 | 53.1 | 49616 |
| F67 | 92003 | 91193 | 98.9 | 95.8 | 53.7 | 50011 |
| F68 | 88851 | 87971 | 99 | 96.3 | 56.4 | 54963 |
| F69 | 85616 | 84854 | 99 | 96 | 53 | 47619 |
| F7 | 91427 | 90737 | 98.7 | 95.3 | 56 | 70336 |
| F70 | 89538 | 88761 | 99 | 96 | 52.3 | 60520 |
| F73 | 90409 | 89531 | 98.9 | 95.9 | 54.4 | 51199 |
| F74 | 90550 | 89935 | 98.9 | 96.1 | 54.2 | 43402 |
| F78 | 91819 | 91024 | 98.8 | 95.6 | 56.6 | 55499 |
| F79 | 85247 | 84363 | 99 | 96.1 | 52.1 | 49250 |
| F81 | 90130 | 89312 | 99 | 96.2 | 54.2 | 63063 |
| F82 | 85469 | 84648 | 99 | 96.2 | 51.3 | 57220 |
| F83 | 85126 | 84250 | 99 | 96.1 | 53.6 | 47964 |
| F84 | 89083 | 88306 | 99 | 96.3 | 51.9 | 41159 |
| F85 | 85759 | 85034 | 99.1 | 96.5 | 52.5 | 65143 |
| F86 | 89055 | 88169 | 98.9 | 96.1 | 53.4 | 49212 |
| F88 | 88550 | 87821 | 99 | 96.2 | 56.1 | 57701 |
| F89 | 86742 | 85964 | 99.1 | 96.4 | 51.9 | 47815 |
| F9 | 86653 | 85672 | 98.9 | 95.9 | 53.6 | 45771 |

Raw total reads: Total number of original sequences sequenced.

Clean total reads: Total number of valid sequences after quality control.

Q20(%): Percentage of bases with sequencing quality score ≥20, indicating sequencing accuracy.

Q30(%): Percentage of bases with a sequencing quality score of ≥30, indicating higher sequencing accuracy.

GC(%): Total proportion of G and C in a DNA sequence.

Sequences number: Final number of sequences obtained after quality control.

**Table S3.** Statistical analysis of soil variables based on Welch’s t-test.

| Soil variations | Elevation | MAT | MAP | | pH | TP | NH_4_^+^ | NO_3_^-^ | SOC | DOC | SUVA_254_ | E_2_/E_3_ | S_R_ |
| --- | --- | --- | --- | --- | --- | --- | --- | --- | --- | --- | --- | --- | --- |
|  | (m) | (°C) | | (mm) |  | (mg/kg) | (mg/kg) | (mg/kg) | (%) | (mg/g) |  |  |  |
| High-latitude mean | 772.79 | 10.87 | | 534.68 | 7.27 | 605.67 | 57.63 | 21.08 | 2.49 | 0.30 | 2.47 | 4.08 | 0.88 |
| Low-latitude mean | 1463.75 | 15.18 | | 1006.42 | 5.64 | 794.28 | 78.42 | 21.38 | 3.29 | 0.53 | 1.43 | 5.11 | 0.94 |
| Lower CI (95%) | -1.06 | -1.20 | | -2.00 | 0.76 | -0.94 | -1.10 | -0.58 | -0.77 | -1.18 | 1.10 | -1.48 | -0.93 |
| Upper CI (95%) | -0.09 | -0.24 | | -0.94 | 1.83 | 0.20 | 0.04 | 0.55 | 0.19 | -0.22 | 2.20 | -0.50 | -0.01 |
| Effect size | -0.58 | -0.72 | | -1.47 | 1.30 | -1.31 | -0.53 | -0.01 | -0.29 | -0.70 | 1.65 | -1.00 | -0.47 |
| P-value | 0.02 | **<0.001** | | **<0.001** | **<0.001** | 0.20 | 0.07 | 0.96 | 0.23 | **<0.001** | **<0.001** | **<0.001** | 0.05 |

The effect size was based on the Hedges’ g model.

MAT: mean annual temperature; MAP: mean annual precipitation; TP: total phosphorus; NH_4_^+^: ammonium nitrogen; NO_3_^-^: nitrate nitrogen; SOC: soil organic carbon; DOC: dissolved organic carbon; SUVA_254_: specific ultraviolet absorbance at 254 nm; E_2_/E_3_: the ratio of the fluorescence signal at 250 nm and 365 nm; S_R_: slope ratio of the 275–295 nm band to that of the 350–400 nm band.

Welch's t-test were tests the null hypothesis that the means of the two groups are equal (H₀: μ₁ = μ₂), against the alternative hypothesis that the means are different (H₁: μ₁ ≠ μ₂).

**Table S4.** Matrix of Pearson correlation coefficients for soil variables.

|  | Elevation | NH_4_^+^ | NO_3_^-^ | TP | AP | MAT | MAP | PH | SOC | DOC | SUVA_254_ | S*R* | E_2_/E_3_ |
| --- | --- | --- | --- | --- | --- | --- | --- | --- | --- | --- | --- | --- | --- |
| Elevation | 1 | -0.3416 | 0.14486 | -0.06188 | -0.1144 | -0.69038 | -0.47185 | 0.329782 | 0.238177 | -0.09451 | 0.113146 | 0.219012 | -0.15116 |
| NH_4_^+^ | -0.3416 | 1 | 0.19682 | 0.221477 | 0.094809 | 0.387755 | 0.473478 | -0.28624 | -0.15431 | 0.227473 | -0.33863 | -0.29762 | 0.17233 |
| NO_3_^-^ | 0.14486 | 0.19682 | 1 | 0.463137 | 0.068891 | 0.036226 | -0.01995 | -0.01961 | 0.051637 | 0.034801 | -0.07654 | 0.017448 | 0.124304 |
| TP | -0.06188 | 0.221477 | 0.463137 | 1 | 0.22005 | 0.22716 | 0.182351 | -0.30619 | 0.047934 | 0.270062 | -0.4548 | -0.02271 | 0.406417 |
| AP | -0.1144 | 0.094809 | 0.068891 | 0.22005 | 1 | 0.239313 | 0.338731 | -0.1221 | -0.08981 | 0.024931 | -0.25502 | 0.268793 | 0.164947 |
| MAT | -0.69038 | 0.387755 | 0.036226 | 0.22716 | 0.239313 | 1 | 0.797366 | -0.53821 | -0.31202 | 0.103008 | -0.50499 | -0.13055 | 0.488226 |
| MAP | -0.47185 | 0.473478 | -0.01995 | 0.182351 | 0.338731 | 0.797366 | 1 | -0.77927 | -0.06452 | 0.359948 | -0.72059 | -0.12036 | 0.478251 |
| PH | 0.329782 | -0.28624 | -0.01961 | -0.30619 | -0.1221 | -0.53821 | -0.77927 | 1 | -0.20138 | -0.56015 | 0.710474 | 0.104496 | -0.53199 |
| SOC | 0.238177 | -0.15431 | 0.051637 | 0.047934 | -0.08981 | -0.31202 | -0.06452 | -0.20138 | 1 | 0.745687 | 0.100769 | -0.12904 | -0.19927 |
| DOC | -0.09451 | 0.227473 | 0.034801 | 0.270062 | 0.024931 | 0.103008 | 0.359948 | -0.56015 | 0.745687 | 1 | -0.23518 | -0.35469 | -0.01988 |
| SUVA_254_ | 0.113146 | -0.33863 | -0.07654 | -0.4548 | -0.25502 | -0.50499 | -0.72059 | 0.710474 | 0.100769 | -0.23518 | 1 | -0.16126 | -0.76521 |
| S*R* | 0.219012 | -0.29762 | 0.017448 | -0.02271 | 0.268793 | -0.13055 | -0.12036 | 0.104496 | -0.12904 | -0.35469 | -0.16126 | 1 | 0.480142 |
| E_2_/E_3_ | -0.15116 | 0.17233 | 0.124304 | 0.406417 | 0.164947 | 0.488226 | 0.478251 | -0.53199 | -0.19927 | -0.01988 | -0.76521 | 0.480142 | 1 |

MAT: mean annual temperature; MAP: mean annual precipitation; TP: total phosphorus; NH_4_^+^: ammonium nitrogen; NO_3_^-^: nitrate nitrogen; SOC: soil organic carbon; DOC: dissolved organic carbon; SUVA_254_: specific ultraviolet absorbance at 254 nm; E_2_/E_3_: the ratio of the fluorescence signal at 250 nm and 365 nm; S_R_: slope ratio of the 275–295 nm band to that of the 350–400 nm band.

**Table S5.** MENs topological properties of different soil groups.

| Soil groups | avgK | avgCC | GD | CD | CS | CB | Con | Trans |
| --- | --- | --- | --- | --- | --- | --- | --- | --- |
| Non-acidic Soil | 10.215 | 0.464 | 5.781 | 0.082 | 20.261 | 0.073 | 0.468 | 0.684 |
| Acidic Soil | 4.675 | 0.342 | 5.376 | 0.03 | 0.443 | 0.039 | 0.164 | 0.513 |
| High-aromaticity soils | 8.691 | 0.396 | 7.441 | 0.106 | 7.489 | 0.19 | 0.52 | 0.539 |
| Low-aromaticity soils | 6.273 | 0.405 | 11.17 | 0.037 | 23.229 | 0.342 | 0.723 | 0.577 |

avgK: average K; avgCC: average clustering coefficient; GD: geodesic distance; CS: centralization of stress centrality; CB: centralization of betweenness; CD: centralization of degree; Trans: transitivity; Con: connectedness.
